# Dendritic polylysine co-delivery of paclitaxel and siAXL enhances the sensitivity of triple-negative breast cancer chemotherapy

**DOI:** 10.3389/fbioe.2024.1415191

**Published:** 2024-08-01

**Authors:** Xiaofeng Wan, Chuanrong Chen, Jianmin Zhan, Shuke Ye, Runsheng Li, Ming Shen

**Affiliations:** ^1^ National Health Commission (NHC) Key Lab of Reproduction Regulation (Shanghai Institute for Biomedical and Pharmaceutical Technologies), Shanghai, China; ^2^ Department of Oncology, Yijishan Hospital of Wannan Medical College, Wuhu, China

**Keywords:** dendritic polylysine, siRNA delivery, tumor microenvironment sensitivity, drug resistance, triple-negative breast cancer

## Abstract

**Background:** Drug resistance is common in triple-negative breast cancer (TNBC) therapy. To identify a method to overcome chemotherapy resistance in TNBC cells, an siRNA targeting the AXL gene (siAXL), which can overcome drug resistance, was used in this study. A nanodelivery system was constructed to co-deliver siAXL and paclitaxel (PTX).

**Methods:** A biodegradable and tumor microenvironment (TME)-sensitive mPEG-coated dendritic polylysine material (PDPLL) was synthesized. This material was used to construct single-molecule nanoparticles to co-deliver PTX and siAXL. The drug encapsulation and morphological properties of the nanoparticles (NPs) were characterized. The sensitivity of the NPs to the TME was evaluated *in vitro* with a dialysis method. The tumor-targeting effect of the PDPLL NPs was evaluated by fluorescence imaging and drug distribution evaluation *in vivo*. The ability to overcome drug resistance was evaluated using PTX-resistant 4T1 cells (4T1/PTX cells) in both *in vitro* and *in vivo* models.

**Results:** PDPLL NPs had a particle size of 49.6 ± 5.9 nm and a zeta potential of 7.87 ± 0.68 mV. The PTX drug loading (DL)% was 2.59%. The siAXL DL was 2.5 mg PDPLL: 10 nmol siAXL. The release of PTX showed sustained release performance. The release of siAXL showed sensitivity for the TME. The NPs were stable in the plasma. The NPs promoted cell uptake by PTX-resistant 4T1 cells (4T1/PTX) and promoted tumor targeting and permeability *in vivo*. siAXL enhanced the toxicity and apoptosis efficiency of PTX in 4T1/PTX cells, as well as the cycle arrest efficiency caused by PTX. The NPs improved the above effects. In mouse 4T1/PTX orthotopic tumors, the NPs enhanced the sensitization of PTX to siAXL.

**Conclusion:** The PDPLL NP co-delivery system possesses good encapsulating potential not only for PTX but also for siRNA. It can enhance the tumor-targeting effect and overcome the drug resistance of 4T1/PTX both *in vitro* and *in vivo*. This system is a potential delivery system for RNAs.

## 1 Introduction

Breast cancer is a malignant tumor with the highest incidence rate among women worldwide. According to statistics from 2021, female breast cancer has surpassed lung cancer as the most common cancer, accounting for 6.9% of the total tumor mortality (Sung et al., 2021) and ranking fifth. In 2022, new cases accounted for approximately 1/4 of new female cancers (Siegel et al., 2022). As of 2023, its incidence rate is still rising at an annual rate of approximately 0.6% (Siegel et al., 2024). The triple-negative breast cancer (TNBC) accounts for 10%–20% of all breast cancers (Morris et al., 2007; Rakha et al., 2007).

TNBC is characterized by strong invasiveness, a poor prognosis, high metastasis, a high mortality rate, and a lack of universal targets. The standard treatment for TNBC is still surgery combined with chemotherapy. Paclitaxel (PTX) is a first-line chemotherapy drug for breast cancer in the clinic. However, its toxicity and drug resistance lead to serious side effect complications (Stordal et al., 2007). Most TNBC patients treated for a short period develop resistance to PTX, leading to a drug-resistant phenotype and the development of malignant progression (Kim et al., 2018). Recent studies have shown that PTX can induce immunogenic cell death (ICD) in tumor cells, triggering specific antitumor immune responses (Su et al., 2020). High-dose PTX is usually associated with severe systemic toxicity, which may lead to immunosuppression (Franzoi and Azambuja, 2020).

AXL receptor tyrosine kinase is significantly upregulated in various human malignant tumors, including TNBC, and has been associated with tumorigenesis and poor prognosis (Zhu et al., 2019). AXL/WRNIP1 mediates the replication stress response and promotes therapy resistance and metachronous metastasis in HER2^+^ breast cancer (Marquez-Palencia et al., 2024). AXL promotes resistance to chemotherapy and tumor stemness and enhances cell invasion by accelerating the epithelial‒mesenchymal transition (EMT) process and forming a positive feedback loop (Antony and Huang, 2017; Konen et al., 2021). Therefore, downregulating the expression of AXL using siRNA (siAXL) may overcome the resistance of PTX. However, siRNAs have poor *in vivo* stability and encounter difficulty during cell uptake (Kara et al., 2022). In our previous research, we used the combination of siAXL and PTX to inhibit TNBC and achieved good results (Chen et al., 2023). In this study, we focused on a new material for improving the efficiency of siAXL delivery.

A nanodrug delivery system (NDDS) needs to perform at least five steps during the delivery process after intravenous injection: blood circulation, accumulation and penetration in the tumor, cellular internalization, and intracellular drug release (Sun et al., 2017). Due to the reduced transcapillary pressure gradient and elevated interstitial fluid pressure (IFP) caused by abnormal tumor vasculature and dysfunctional lymphatic drainage (Ju et al., 2014; Nia et al., 2020; Souri et al., 2022), it is difficult for large NDDSs (>100 nm) to penetrate deep into tumor sites. NDDSs with small particle sizes (especially those less than 30 nm) have been demonstrated to penetrate poorly permeable tumors (Ikeda-Imafuku et al., 2022).

Polylysine (PLL) is a type of cationic polymer. PLL and its derivatives have been used as carriers for nucleic acids (Kim et al., 2023). However, delivery systems assembled with linear materials have the shortcomings of larger particle sizes, leading to poor tissue permeability, as well as corrosion, leading to drug leakage.

Dendrimers are prepared with a level of control not attainable with most linear polymers, leading to nearly monodisperse globular macromolecules with many peripheral groups. Consequently, dendrimers are ideal delivery vehicle candidates for nanodelivery systems (Wolinsky and Grinstaff, 2008; Mendes et al., 2017). Dendritic polylysine (DPLL) is a dendrimer of polylysine that can adsorb nucleic acids with positive charges on the surface and internally. It can also form monomolecular nanoparticles with the advantages of a stable structure, small particle size, and strong penetration ability. Compared with linear polymers, it is a better carrier material for transporting nucleic acid substances (Byrne et al., 2013).

The positive charge on the surface of DPLL also leads to toxicity and is easily captured by the complement system in the carrier cycle, resulting in poor tumor targeting. mPEG is commonly used to improve its biocompatibility and increase long-term cycling ability (Barrios-Gumiel et al., 2020). Microenvironmentally sensitive bonds are used to connect mPEG and DPLL so that the nanoparticles maintain stability in body circulation (Chen et al., 2021; Shen et al., 2022). mPEG can detach at the tumor site, followed by uptake of the NPs by TNBC cells.

**FIGURE 1:**
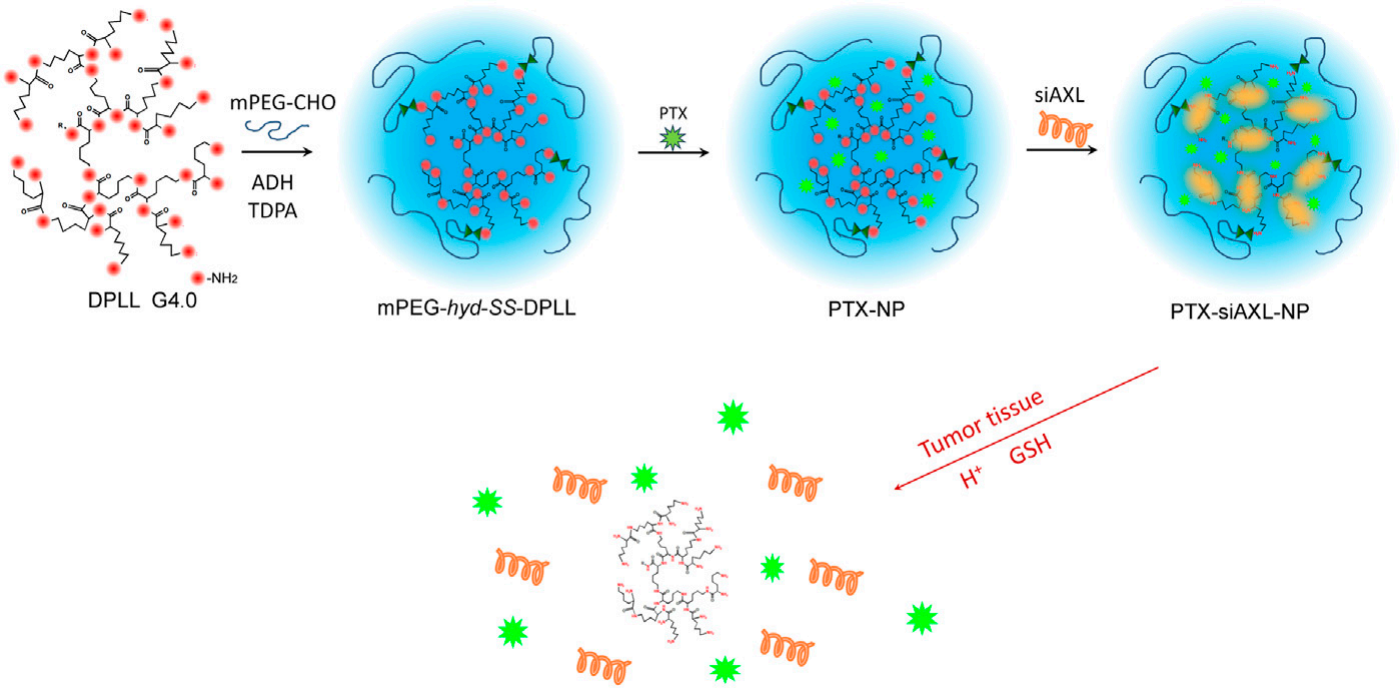
Scheme of the PDPLL co-delivery system.

The tumor microenvironment (TME) has a low pH and high glutathione (GSH) concentration (Tannock and Rotin, 1989; Engin et al., 1995; Ojugo et al., 1999; Estrela et al., 2006). The use of environmentally sensitive bonds to connect PEG can cause it to detach when the NPs reach the tumor tissue (Chen et al., 2021; Shen et al., 2022). Therefore, in this research, an intelligent drug delivery system was formed by connecting mPEG via a pH-responsive hydrazone and a GSH-responsive disulfide bond on the surface of DPLL to obtain an mPEG-covered DPLL (PDPLL). PTX and siAXL were co-delivered to overcome tumor cell resistance (Figure 1). The environmental sensitivity, tumor targeting, and antitumor efficacy were evaluated *in vitro* and *in vivo*.

## 2 Materials and methods

### 2.1 Materials

DPLL was purchased from Shandong Weihai Chenxi Chemical Co., Ltd. 4-Dimethylaminopyridine (DMAP), 3,3′-dithiodipropionic acid (TDPA), triethylamine, N-(3-dimethylaminopropyl)-N′-ethylcarbodiimide hydrochloride (EDC), 1-hydroxybenzotriazole (BtOH), and PTX were purchased from Sigma‒Aldrich (St. Louis, United States). All siRNAs were purchased from Ruibo Biotechnology, Inc. (Guangzhou, China). The sequence of siAXL was sense 5′-GGA​GAC​CCG​UUA​UGG​AGA​ATT-3′ and antisense 5′-UUC​UCC​AUA​ACG​GGU​CUC​CTT-3′. The AXL primers for q-PCR (QM24450S), MTT (ST316), Annexin V-FITC (C1062L), Cell Cycle and Apoptosis Analysis Kit (C1052), BCA Protein Assay kit (P0011), and Lipo6000 (C0526) were purchased from Beyotime Biotechnology, Inc. (Shanghai, China). The antibodies of BCl-2 (AG1222), BAX (AF0057), and β-actin (AA128) for Western blot assay were purchased from Beyotime Biotechnology, Inc. (Shanghai, China). MMP-9 antibody (ab38898) was purchased from Abcam, Inc. (English). The vimentin antibody (5741S) was purchased from Cell Signaling Technology, Inc. (United States). PTX-resistant 4T1 cells (4T1/PTX) were generated in our laboratory (Chen et al., 2023). Female BALB/c mice were obtained from Shanghai Lingchang Biotechnology Co., Ltd. (Shanghai, China). The animal experiments were performed under protocols approved by the Animal Care and Use Committee of the Shanghai Institute for Biomedical and Pharmaceutical Technologies (Ethics number 2021-05).

### 2.2 Synthesis of the mPEG-hyd-SS-DPLL material

A portion of 0.5 g of mPEG was dissolved in pure water. A portion of 37 mg adipic dihydrazide (ADH) was added. The mixture was stirred at room temperature for 24 h. Then, it was dialyzed in water to remove small molecules and collect mPEG-ADH.

The mPEG-ADH was dissolved in dry CHCl_3_. Thiodipropionic acid (TDPA), dicyclohexylcarbodiimide (DCC), and 4-dimethylaminopyridine (DMAP) were added at a molar ratio of 1:2:2 to the solution of mPEG-ADH. Nitrogen was injected with stirring to remove oxygen for 30 min. The mixture was stirred overnight at room temperature. The product was dialyzed in CH_2_Cl_2_ to remove small molecules and dried under vacuum to obtain mPEG-*hyd*-COOH.

The mPEG-*hyd-SS*-COOH, DPLL G4.0, 1-(3-dimethylaminopropyl)-3-ethylcarbodiimide hydrochloride (EDC), and 1-hydroxybenzotriazole (BtOH) were dissolved in water separately at a molar ratio of 4:1:8:8. The four solutions were subsequently mixed and stirred under nitrogen to remove oxygen for 30 min. The mixture was stirred overnight at room temperature. Then, it was dialyzed and lyophilized to obtain mPEG-*hyd*-*SS*-DPLL (abbreviated as PDPLL).

### 2.3 Preparation and morphological characterization of PDPLL nanoparticles

#### 2.3.1 Encapsulation of PTX

PDPLL was dissolved in an acetonitrile solution containing PTX. The solution was injected into pure water with stirring for 5 more minutes. The acetonitrile was removed by vacuum rotary evaporation to obtain PTX-NPs. The total PTX content in the PTX-NP solution was determined by high-performance liquid chromatography (HPLC, Agilent 1100, United States) after being diluted 10 times with acetonitrile (C_0_). The PTX was determined with C_18_ HPLC column and a mixture of acetonitrile −10 mmol/L NH_4_Ac solution (the pH value was adjusted to 5.0 with H_3_PO_4_) 53:47 as the mobile phase. The column temperature was 30°C. The flow rate was 1 mL/min. PTX peak was determined at the wavelength of 227 nm. Free PTX was separated by centrifugation at 5,000 rpm for 5 min. The PTX concentration in the supernatant (C_1_) was determined to be equal to that in the PTX-NPs (C_0_). The encapsulation efficiency and drug loading were calculated as follows.

$$\text{Encapsulation efficiency }\%=C_{1}/C_{0}*100\%.$$

$$\text{Drug loading }\%=C_{1}/\text{Weight}\text{ of NP}*100\%.$$

#### 2.3.2 Encapsulation of siAXL

siAXL was added to PTX-NPs at different ratios with stirring for 2 h to obtain PTX-siAXL-NPs. The free siAXL was separated by agarose electrophoresis. The spots were subjected to grayscale integration by ImageJ software, and the encapsulation efficiency and drug loading were calculated. The particle size and potential of the NPs were characterized using a particle size meter (Marlvern ZS, Egland). The particle morphology was characterized using transmission electron microscopy (Talos L120C G2, Thermo Fisher, United States).

### 2.4 Microenvironmentally sensitive release of PTX and siAXL controlled by PDPLL NPs

The release of the NPs was evaluated using a dialysis method. The media used were PBS containing 2 μM, 2 mM, or 20 mM, and the pH was 5.0, 6.5, or 7.4, respectively. A group of mouse plasma samples was also investigated.

For PTX, 1 mL of PTX-NPs was sealed in a dialysis bag (MWCO = 3,500) and then immersed in 20 mL of release media with different pH values and GSH concentrations. The mixture was released with shaking at 37°C. A 0.2 mL portion was removed at a set time for HPLC determination. An equal volume of the same medium was added at the same time. The PTX concentrations were calculated, and the cumulative release curves were generated and compared with those of the PTX solution.

For siAXL, 1 mL of Cy5-siAXL-NPs was sealed in a dialysis bag (MWCO = 100,000) and then immersed in 10 mL of release media with different pH values and GSH concentrations. The mixture was released with shaking at 37°C. A portion of 1 mL was removed at the set time to determine the Cy5 fluorescence intensity with a spectrophotometer (Hitachi F-2700, Japan). An equal volume of the same medium was added at the same time. The siAXL concentrations were calculated, and cumulative release curves were generated and compared with those of the siAXL solution.

### 2.5 Uptake by 4T1/PTX cells

Coumarin-6 was adsorbed with PDPLL to obtain Coumarin-6-NP. 4T1/PTX cells (3*10^5^ per well) were seeded in six-well plates overnight and then treated with free coumarin-6 and coumarin-6-NP for 5 h. The cells were washed with cold PBS twice and fixed with 4% paraformaldehyde solution for 15 min. The cell nucleus was stained with Hoechst for 5 min. The fluorescence was observed using a fluorescence microscope (Olympus SZX7, Japan).

### 2.6 Cytotoxicity and compatibility

4T-1/PTX and 4T1 cells were used for cytotoxicity experiments. The L929 cells were used for cytocompatibility. The cells (2*10^4^ per well) were seeded in a 96-well plate and incubated overnight. The drug solutions and NPs were added to the culture medium with a 1 μg/mL concentration of PTX and a 100 pmol/mL concentration of siAXL. The PTX solution was prepared with dimethyl sulfoxide and diluted with the culture medium. siAXL was transfected into cells with Lipo6000, and the culture medium was changed after 5 h. The NPs were added to the cells directly. The drug-free PDPLL NPs were diluted with the culture medium. After 48 h, the culture media were replaced with 0.2 mL culture media containing 0.5 mg/mL MTT. The cells were incubated for another 2–4 h. The culture media were discarded. The residues were dissolved in 0.2 mL of DMSO with shaking in the dark for 20 min. The absorbance at 490 nm was measured with a microplate reader (Thermo Multiskan MK3, United States). The relative proliferation rate was calculated as follows:

Relative proliferation rate %=(A‐sampleA)zero/(A‐controlA)zero*100%.

### 2.7 NP-induced apoptotic and cycle arrest effects on 4T1/PTX cells

4T1/PTX cells (3*10^5^ per well) were seeded in a 12-well plate and incubated to adhere to the bottom. The NPs or drugs were added to the culture for 24 h. The cells were subsequently digested with trypsin and collected by centrifugation. Cytoapoptosis was determined by flow cytometry (BD LSRFortessa, United States) after Annexin V and PI staining. The cell cycle distribution was evaluated by flow cytometry after PI staining.

### 2.8 *In vivo* tumor-targeting effect

4T1/PTX cells were digested, centrifuged, and resuspended in PBS. The cell suspension was injected (5 × 10^5^ cells/mouse) into the right upper breast of each mouse (female Balb/c mice, 6–8 weeks old) to establish an orthotopic breast tumor model. After 10 days, DIR-NPs and DIR solution were injected into the tail vein separately. Observations were performed with an *in vivo* imaging system (Putzmeister VSIQUE *In vivo* Smart-LF, Germany) at 1, 4, 24, and 48 h after injection. The mice were sacrificed at the 48 h [the mice were anaesthetized with isoflurane (1%–1.5%)]. After the animals were completely anesthetized, they were euthanized by cardiac bleeding. Important organs and tumors were removed to detect the fluorescence distribution. The tumors were then frozen and sliced to observe the fluorescence within the tumor using a PANNORAMIC SCAN II Digital Slide Scanner (3DHISTECH, Hungary).

Pharmacokinetic evaluation and simple tissue distribution experiments were conducted in mice. The mice were injected with PTX (1 mg/kg) or cy5-siAXL solution (100 nmol/kg) via the tail vein. Blood was collected from the eye socket at set time points. The blood was centrifuged, and the plasma was separated. The drug contents were measured, and drug‒time curves were plotted (n = 6). Another group of orthotopic breast tumor-bearing mice was euthanized at 24 and 48 h. Important organs and tumors were removed. The organs and tumors were washed and homogenized. The drug contents were measured, and the drug tissue distribution was determined (n = 6). The PTX content was determined by LC-MS using a previously reported method (Shen et al., 2011). Cy5-siAXL was determined and integrated with the plasma and homogenate using an *in vivo* imaging system.

### 2.9 *In vivo* antitumor effects

The orthotopic 4T1/PTX tumors of the mice were set up as described in Section 2.8. The tumor volumes were measured with a Vernier Calliper and calculated as πab^2^/6 (a: the longest diameter and b: the longest diameter vertical to a). When the tumors grew to 50–70 mm^3^, the mice were randomly divided into five groups. Normal saline, PTX solution (0.5% F-68), PTX + siAXL solution, PTX-NPs, and PTX-siAXL-NPs were injected into the tail vein separately at a PTX dose of 1 mg/kg and a siAXL dose of 100 nmol/kg. The injections were performed once every 3 days for five times. The body weight and tumor volume were measured before every injection. After five injections, the mice were killed [the mice were anesthetized with isoflurane (1%–1.5%)]. After the animals were completely anesthetized, they were euthanized by cardiac bleeding. The tumors and important organs were removed. The tumors were weighed to compare the therapeutic effects. The organs were embedded in paraffin and sliced for pathological analysis.

### 2.10 Western blot analysis

4T1/PTX cells were treated with different preparations as described in Section 2.7. Then, the cells were washed with PBS twice to remove exogenous proteins, and total protein was extracted. Western blotting was performed using standard procedures. Briefly, total protein (20 μg/well) was separated on SDS‒PAGE gels and transferred to PVDF membranes by electroblotting. These membranes were incubated with primary antibodies against BCl-2 (Beyotime, AG1222), BAX (Beyotime, AF0057), MMP-9 (Abcam, ab38898), vimentin (CST, 5741S), and β-actin (Beyotime, AA128) at 4°C overnight and then incubated with HRP-conjugated anti-mouse/rabbit secondary antibodies (Abcam, ab205719; CST, 7074S) for 1 h at room temperature. The protein bands were visualized using a Bio-Rad imaging system (Bio-Rad, United States). The expression levels of the proteins were quantified using Image Lab software (version 3.0; Bio-Rad, United States).

### 2.11 q-PCR detection of AXL mRNA in 4T1 and 4T1/PTX cells

4T1/PTX cells and 4T1 cells (1.5*10^5^ per well) were seeded in 24-well plates. Cells were treated with Lipo6000+siNC, Lipo6000+siAXL, or siAXL-NP. The cells were subsequently collected and treated according to the protocol. q-PCR was conducted with Genious 2X SYBR Green Fast q-PCR Mix (ABclonal, CAT# RK21205), using a LightCycler 480 II device (Roche).

### 2.12 P-glycoprotein determination by ELISA

To evaluate the effect of siAXL on the drug resistance of 4T1/PTX cells, P-glycoprotein (P-gp) levels were measured with an ELISA kit. The cells were treated as described in Section 2.11 and were then collected. The P-gp content was determined according to the protocol. The total protein concentration of the cells was determined with a BCA protein assay kit. The ratios of the P-gp content to the total protein content were then calculated.

### 2.13 Statistical analysis

The data were analyzed with Excel software and are presented as the means ± SD. Student’s t-test and one-way analysis of variance (ANOVA) were performed to compare differences between groups. *p < 0.05 and **p < 0.01 were considered statistically significant.

## 3 Results and discussions

### 3.1 PDPLL NP characterization

Lysine polymers possess good tissue biocompatibility and are widely used as biocompatible materials in tissue engineering (Yuan et al., 2018). Due to its high-positive charge, its loading capacity for nucleic acids is high. However, the positive charge is toxic to cells. In addition, due to their positive charges, PLL NPs are easily captured by the negatively charged complement system in the body, leading to poor targeting (Hatakeyama et al., 2013). Therefore, we modified its surface with the water-soluble polymer mPEG. However, PEG inhibits cellular uptake, which is known as the PEG dilemma. Although the NPs are distributed more in tumor tissue, their uptake by tumor cells does not increase. Acid-sensitive hydrazone bonds and GSH-sensitive disulfide bonds were used to connect mPEG and DPLL. In the acidic TME with a high GSH concentration, the detachment of mPEG was doubled. The results showed that the NPs not only possess a high loading efficiency of siAXL but also promote uptake in PTX-resistant cells. The release behavior of siAXL was acid- and GSH-sensitive, which supports our design.

**FIGURE 2:**
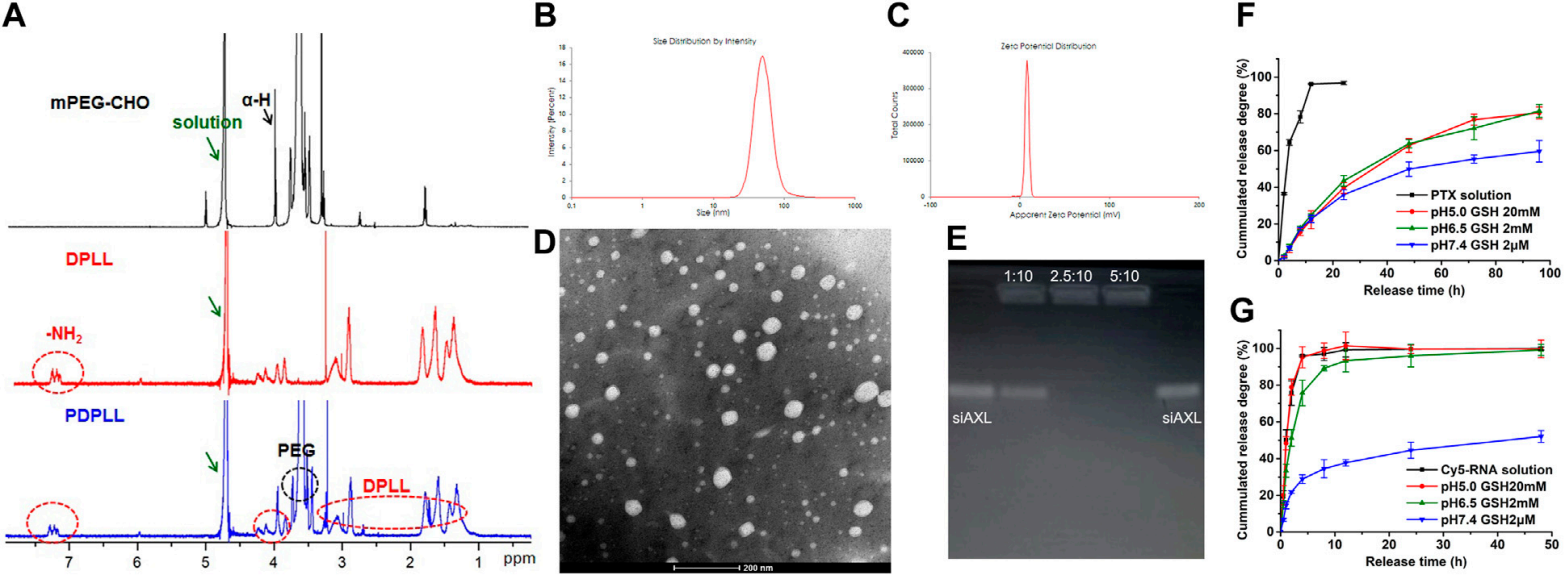
Characterization of the PDPLL material and the NPs. **(A)** ^1^H NMR spectrum of the PDPLL material. **(B,C)** Particle size and zeta potential of PDPLL NPs. **(D)** Electron micrograph of the NPs after phosphotungstic acid staining. **(E)** Agarose gel electrophoresis characterization of siAXL encapsulated at different ratios of PDPLL NPs (mg) to siAXL (nmol). **(F,G)** PTX and siAXL release behaviors from NPs in media with different pH values and GSH concentrations.

The PDPLL material was successfully synthesized (Figure 2A). The particle size of the nanoparticles was 49.6 ± 5.9 nm, and the zeta potential was 7.87 ± 0.68 mV. The particles were nearly spherical as observed by electron microscopy (Figures 2B–D). The drug-loading efficiency of PTX was 2.59%, with an encapsulation efficiency of 84.69%. siALX was completely adsorbed at a ratio of 2.5 mg PDPLL:10 nmol siALX (Figure 2E).

The PTX release behavior revealed poor TME sensitivity, possibly because of its lipophilicity (Figure 2F). However, the release rate of siAXL was accelerated in acidic media with high GSH concentrations. In contrast, the release was slow in pH 7.4 medium containing 2 μM GSH and mouse plasma, which was similar to the normal physiological environment *in vivo* (Figure 2G). These parameters could improve the *in vivo* distribution and efficacy of siAXL.

### 3.2 *In vitro* antitumor evaluation of the NPs

**FIGURE 3:**
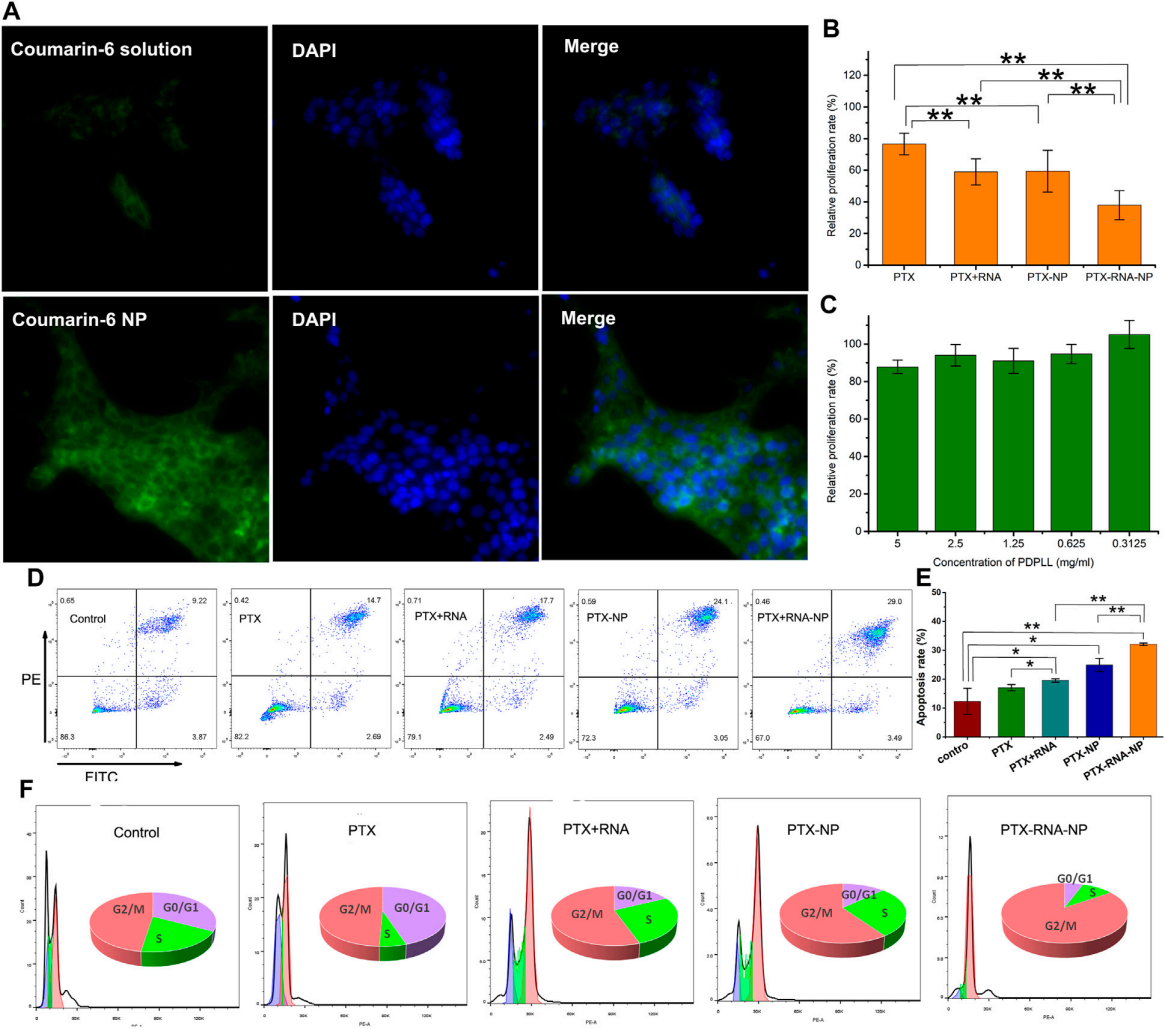
Evaluation of the tumor-targeting and antitumor effects of PDPLL NPs on 4T1/PTX and 4T1 cells. **(A)** Increased fluorescence uptake in 4T1/PTX cells by NPs. **(B)** Growth inhibition of 4T1/PTX and 4T1 cells after different treatments. **(C)** Compatibility of the PDPLL material in L929 cells. **(D,E)** 4T1/PTX cell apoptosis was increased after different treatments. **(F)** Cycle arrest effects of different treatments on 4T1/PTX cells. *p < 0.05 and **p < 0.01.

Coumarin-6 fluorescence was weak in 4T1/PTX cells. However, it increased significantly when coumarin-6 was encapsulated by PDPLL (Figure 3A), indicating that the uptake by 4T1/PTX cells was increased significantly by the NPs.

The cytotoxicity of PTX to 4T1/PTX cells was significantly enhanced by encapsulation of the nanoparticles. Moreover, siAXL significantly increased the toxicity of PTX either in solution or in the NP state. In contrast, the toxicity to 4T1 cells was quite different. The PTX solution exhibited strong toxicity, whereas the NPs did not have a good inhibitory effect because their uptake speed was slower than that of the free drug. siAXL did not promote cytotoxicity in 4T1 cells. Overall, there was no significant difference in the results between free drugs and nanoparticles in 4T1 cells (Figure 3B). The Lipo6000 control group showed no toxicity to the two cell lines. These results confirmed the ability of NP and siAXL to overcome drug resistance.

The results of the drug-free NPs showed that the relative proliferation rate of L929 cells was greater than 80% at a concentration of 5 mg/mL, which indicated that PDPLL possesses good cell compatibility due to the mPEG block (Figure 3C). Along with the results of the TME-sensitive release properties evaluated above, the PDPLL material has achieved the aim of the design.

The results of the apoptosis analysis were similar to those of cytotoxicity in 4T1/PTX cells. The level of apoptosis in 4T1/PTX cells treated with PTX-NPs was significantly greater than that in those treated with PTX alone. siAXL significantly increased PTX-induced apoptosis both in solution and in the NP state (Figures 3D, E). PTX blocked 4T1/PTX cells in the G2/M phase. Both the NPs and siAXL promoted cell cycle arrest (Figure 3F).

### 3.3 *In vivo* tumor-targeting evaluation of the NPs

**FIGURE 4:**
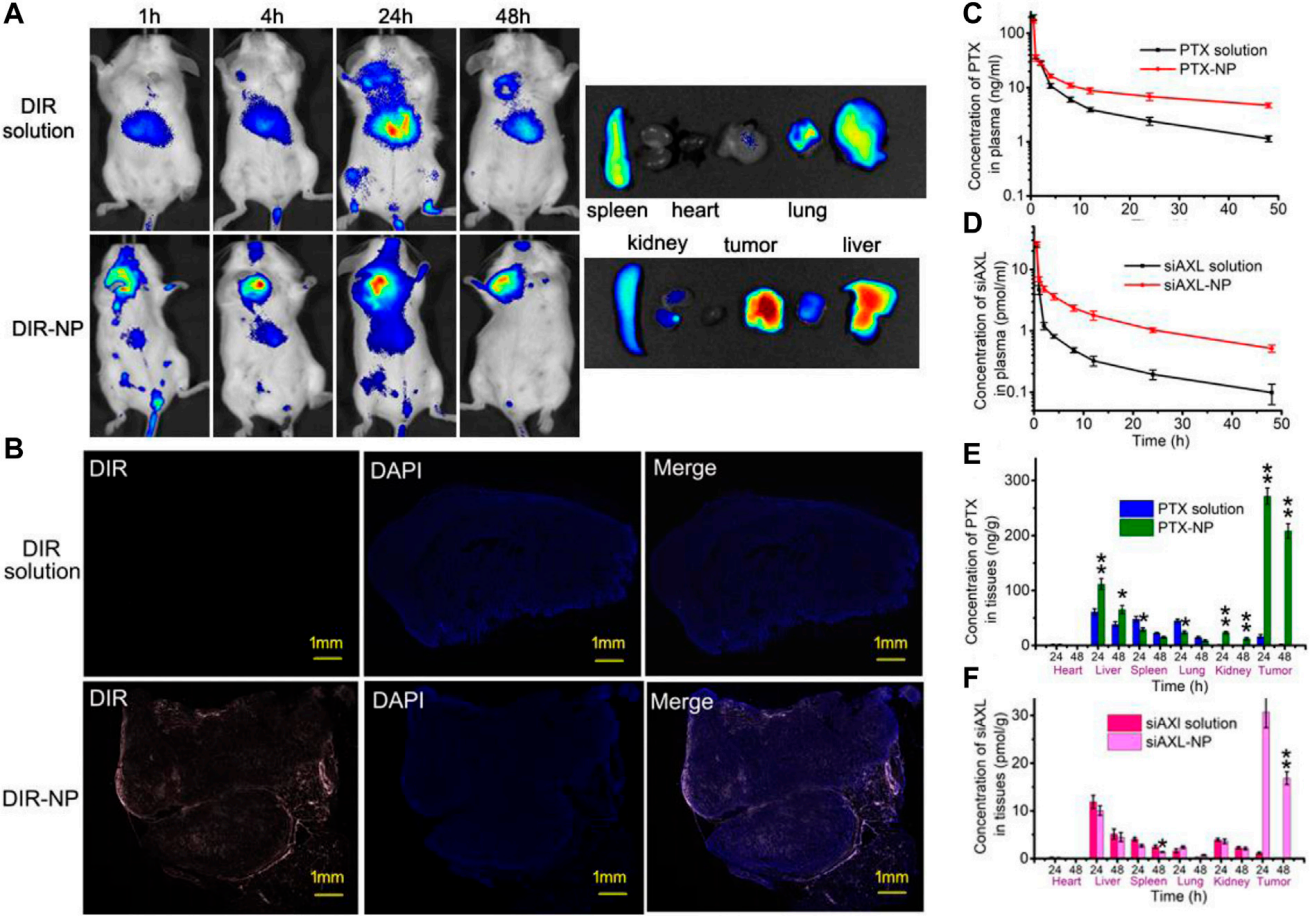
*In vivo* tumor-targeting effect of PDPLL NPs. **(A)** Distribution of DIR fluorescence in 4T1/PTX cell tumor-loaded mice by *in vivo* imaging. **(B)** Distribution of DIR in tumors in frozen sections. The red fluorescence indicates DIR, and the blue fluorescence indicates DAPI. **(C,D)** Concentrations of PTX and cy5-siAXL in the plasma of mice at different times after injection with drug solution or NPs. **(E,F)** The contents of PTX and Cy5-siAXL in the heart, liver, spleen, lung, kidney, and tumor of mice at 24 h and 48 h after injection with drug solution or NPs. *p < 0.05 and **p < 0.01.

The results of *in vivo* imaging (Figure 4A) revealed that the fluorescence of the DIR solution group was distributed mainly in the livers of BALB/c mice bearing 4T1/PTX orthotopic tumors, but was low in the tumors. In the DIR-NP group, the fluorescence in the tumor was much stronger. A strong fluorescence signal could still be observed at the tumor site 48 h after injection. The organ fluorescence signals were similar, indicating that the NPs had strong tumor-targeting ability.

Fluorescence imaging of the tumor slides revealed that the red fluorescence of DIR was almost not visible in the tumors of the DIR solution group. In contrast, fluorescence in the tumors of the DIR-NP group penetrated the interior of the tumor, indicating strong permeability of the NPs in the tumor tissue (Figure 4B).

The distributions of PTX and siAXL were also investigated. The PTX and Cy5-siAXL concentrations in mouse plasma decreased more slowly after injection with the NPs than after injection with the drug solutions, which confirmed the long circulation capacity of the NPs (Figures 4C, D). The distributions of PTX and cy5-siAXL in important organs were determined at 24 h and 48 h after injection. As shown in Figures 4E, F, neither PTX nor cy5-siAXL was enriched in the heart. The concentration of PTX was significantly greater in the liver and kidney, especially in the tumor itself, while those values were decreased in the spleen and lung. This finding indicates that the NPs changed the biodistribution of the drug and the perfect permeability of the tumor to the PDPLL NPs. For cy5-siAXL, the concentration in the tumor was remarkably higher. The distributions in the important organs were not significantly different from those in the spleen at 48 h (Figure 4F). This may be due to the water solubility of siRNA, which leads to rapid excretion in the organs after siAXL is released from the NPs. In contrast, the irregular blood vessels in tumors make their excretion difficult and exacerbate enrichment in the tumor.

Dendrimers can form monomolecular nanoparticles. The drugs are adsorbed by functional groups on the surface or encapsulated in internal holes. Due to their chemical stability, the particles formed can remain stable during circulation, avoiding drug leakage to some extent. They have better permeability in tissue because of their small volume, which was also demonstrated in the fluorescence distribution results of the tumor slices. The tumor-targeting ability and permeability of the PDPLL NPs *in vivo*, as well as the sensitive drug release behavior of the TME, demonstrate the ability of the PDPLL NPs to overcome tumor inhibition and drug resistance *in vivo*.

### 3.4 *In vivo* therapeutic efficacy evaluation in 4T1/PTX tumor-bearing mice

**FIGURE 5:**
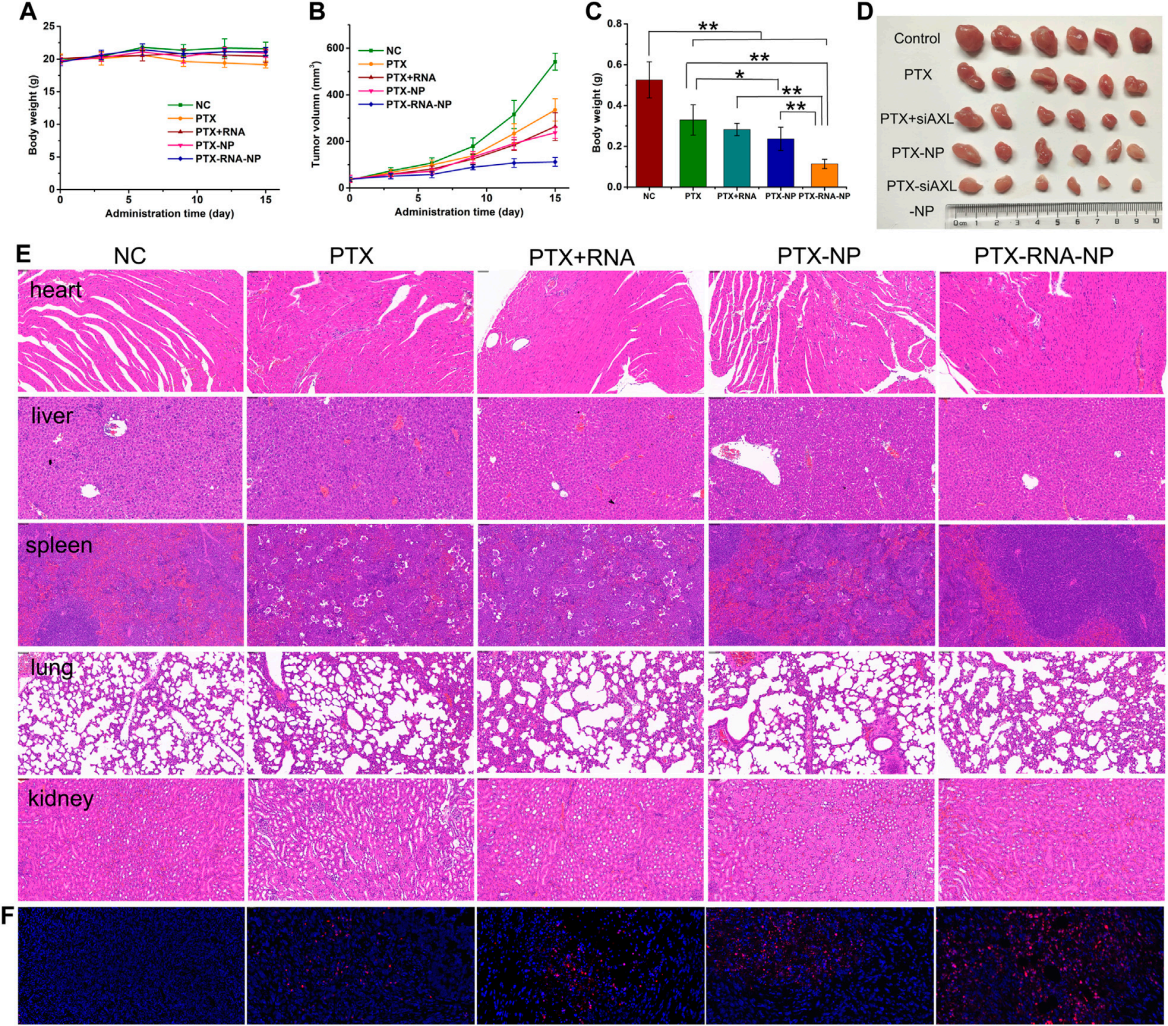
*In vivo* antitumor effects in 4T1/PTX orthotopic tumor-bearing mice. **(A)** Mouse body weight during the treatment process. **(B)** Tumor growth curve during the treatment process. **p < 0.01 compared with the control group. **(C)** Tumor inhibition rates of the treatment groups. *p < 0.05 and **p < 0.01. **(D)** Tumor images of the treatment groups. **(E)** H&E staining results of heart, liver, spleen, lung, and kidney tissues. **(F)** TUNEL staining results of tumor tissues from the treatment groups.

After five treatments, there was no significant change in body weight, except for a slight decrease in the PTX group in the later stage of the experiment (Figure 5A). Compared with those in the control group, the tumor size and weight in the PTX, PTX + siAXL, and PTX-NP groups were significantly lower. Compared with those of the PTX group, the tumor size and weight of the PTX-NP group were significantly lower. The tumors of the PTX-siAXL-NP group were the smallest, and the differences were significant compared to the PTX, PTX + siAXL, and PTX-NP groups. However, there was no significant difference between the PTX + siAXL group and the PTX group (Figures 5B–D). These results indicate that PTX has a therapeutic effect on 4T1/PTX cells in orthotopic tumors and that the encapsulation of the NPs promoted the therapeutic effect of PTX. The solution form of siAXL did not have any effect on overcoming PTX resistance *in vivo*. However, the resistance effect was remarkably overcome after encapsulation by the NPs.

The H&E results revealed slight atrophy in the spleen tissues of the PTX group and PTX + siAXL group, whereas no significant toxic reactions were observed in the other groups (Figure 5E). The TUNEL staining results revealed that the degree of internal apoptosis in the tumors was similar to the tumor size and weight results (red florescence, Figure 5F).

Combined with the *in vitro* results, the effective delivery of siAXL is highly important for its *in vivo* applications. The PDPLL NP is a good delivery agent for siAXL.

### 3.5 The molecular mechanism by which siAXL improves the antitumor efficacy of PTX in 4T1/PTX cells

The resistance of PTX is related to the expression of several proteins and RNAs (Li et al., 2018; Liao et al., 2023; Zhao et al., 2024). AXL receptor tyrosine kinase is significantly upregulated in various human malignant tumors, including TNBC, and has been associated with tumorigenesis and poor prognosis (Antony and Huang, 2017; Tsukita et al., 2019; Zhu et al., 2019; Konen et al., 2021; Maimon et al., 2021). The expression of siAXL was correlated with that of P-glycoprotein, indicating the multidrug-resistant nature of tumor cells (Zhao, et al., 2012). PTX resistance can be overcome by siRNA targeting AXL (Chen et al., 2023).

**FIGURE 6:**
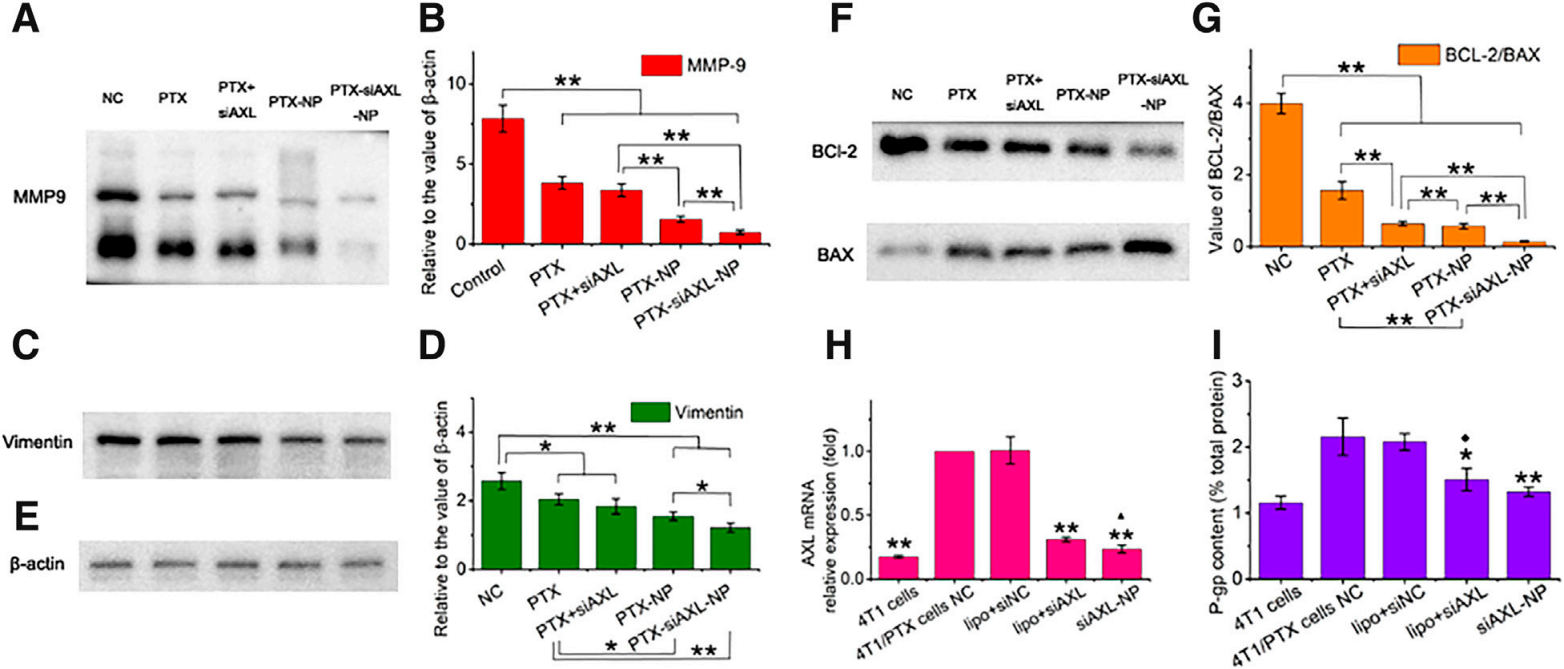
Expression levels of EMT- and apoptosis-related proteins or genes in 4T1/PTX cells treated with PTX and siAXL preparations. **(A,B)** Western blot results for the levels of MMP-9, **(C,D)**, vimentin, **(E)**, and β-actin. **(F,G)**, BCL-2 and BAX expression in 4T1/PTX cells after different treatments for 48 h *p < 0.05 and **p < 0.01. **(H)** PCR results for the relative levels of 4T1/PTX cells after different treatments and the original 4T1 cells. **p < 0.01 compared with 4T1/PTX NC cells, ▲*p* < 0.05 compared with 4T1/PTX cells treated with siAXL and transfected with Lipo6000. **(I)** P-gp levels in 4T1/PTX cells after different treatments and in the original 4T1 cells. *p < 0.05 and **p < 0.01 compared with the 4T1/PTX cell NC, ◆*p* < 0.05 compared with the original 4T1 cells.

As shown in Figure 6H, after siAXL transfection, the AXL mRNA levels in 4T1/PTX cells were significantly reduced, regardless of whether the cells were transfected with Lipo6000 or PDPLL NPs. The transfection efficiency of the PDPLL NPs was significantly greater than that of Lipo6000. As expected, after transfection with siNC, the AXL mRNA level was almost unchanged. As shown in Figure 6I, the level of P-gp, which is a characteristic of drug resistance, also significantly decreased in 4T/PTX cells after transfection with siAXL. However, there was no significant change after the cells were transfected with siNC. There was no significant difference between the levels of P-gp after NP transfection and Lipo6000 transfection. However, compared with the P-gp level in original 4T1 cells, a significantly greater P-gp level was detected in 4T1/PTX cells transfected with Lipo6000, whereas no significant difference was detected in those transfected with NPs. These findings indicate that the PDPLL NPs can effectively transfect siAXL into 4T1/PTX cells better than Lipo6000 can.

The results of PCR analysis of siAXL in 4T1/PTX cells revealed that AXL was significantly downregulated. AXL mRNA expression was somewhat reduced to the level in 4T1 cells when siAXL was delivered by PDPLL NPs. A similar trend was found for P-gp expression, which indicates multidrug resistance. However, significant tumor inhibition effects were observed only for the PDPLL NPs *in vivo*, which also confirmed the importance of the stability and targeting of siAXL delivery *in vivo*.

β-actin was used as the loading control (Figure 6E). The protein expression in 4T1/PTX cells treated with PTX, PTX + siAXL, PTX-NP, or PTX-siAXL-NP changed significantly. The expression of MMP-9 and vimentin was downregulated significantly, indicating the inhibition of TNBC metastasis by PTX together with siAXL. The PDPLL NP encapsulation enhanced the inhibitory effect on metastasis (Figures 6A–D).

The expression of BCl-2 and BAX is related to apoptosis. The ratio of BCL-2 to BAX was decreased after PTX treatment. Transfection with siAXL exacerbated this downregulation. NP encapsulation promoted this downregulation in both the PTX-NP and PTX-siAXL-NP groups. Moreover, siAXL enhanced the downregulation of PTX-NPs when they were encapsulated together (Figures 6F, G). This result verified the ability of PTX-siAXL-NP to promote apoptosis.

## 4 Conclusion

In summary, the environmentally sensitive PEG-coated dendritic polylysine used in this study as a carrier for the co-delivery of PTX and siAXL can overcome TNBC resistance to PTX and improve chemotherapeutic efficacy. PDPLL can be used as an excellent carrier material for delivering RNA drugs.

## Data Availability

The original contributions presented in the study are included in the article/Supplementary Material; further inquiries can be directed to the corresponding author.
