# Supplementary material for: Dendritic polylysine co-delivery of paclitaxel and siAXL enhances the sensitivity of triple-negative breast cancer chemotherapy
Source: Front Bioeng Biotechnol. 2024 Aug 1;12:1415191. doi: 10.3389/fbioe.2024.1415191 (PMC11324506; doi:10.3389/fbioe.2024.1415191)
Supplement: Supplementary file 1 [file DataSheet1.PDF]

Supplentary material: photos of WB in Figure 6

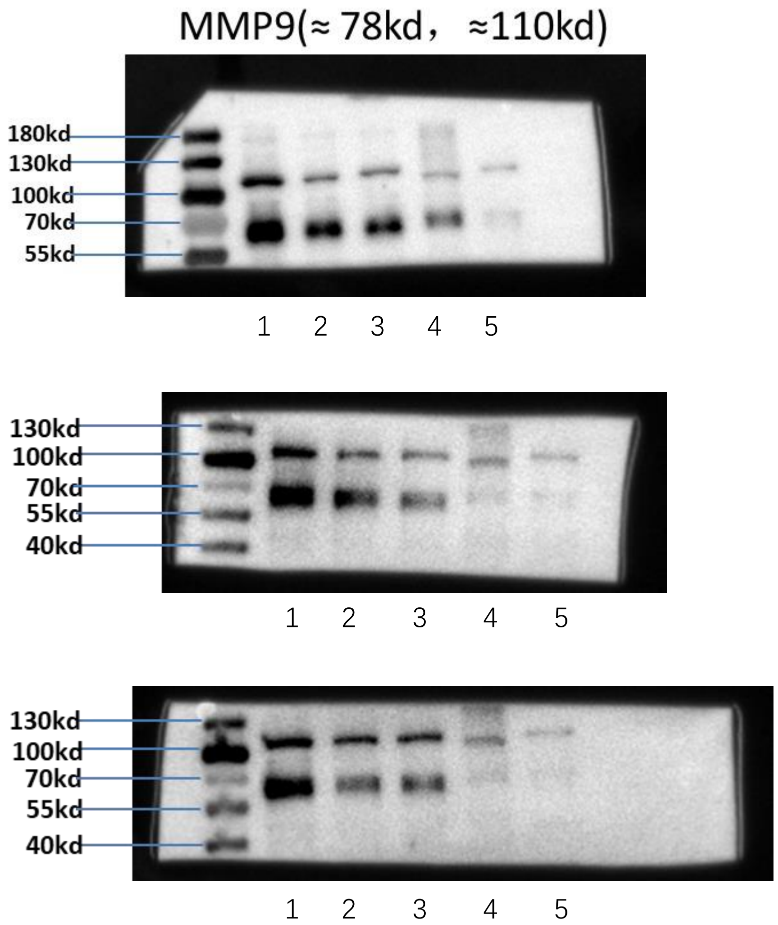

Figure S1 The WB raw photo of MMP -9 in Figure 6. 1~5: NC, PTX, PTX+siAXL, PTX-NP, PTX-siAXL-NP

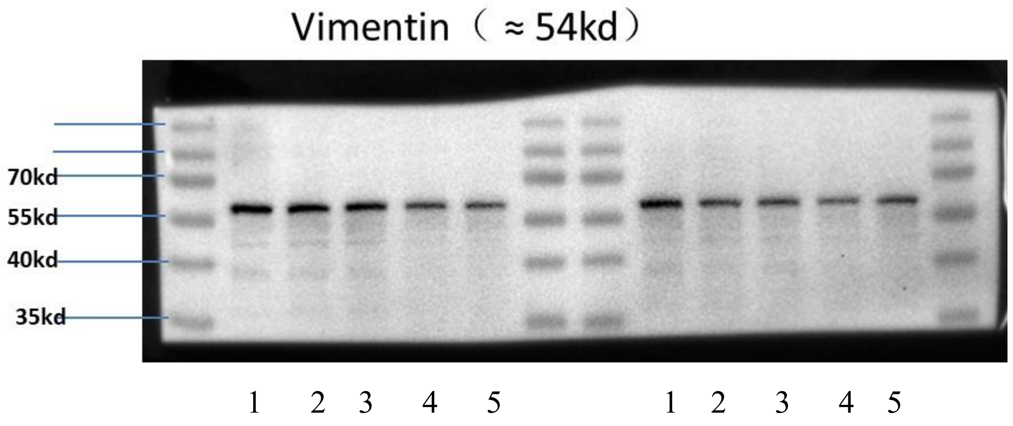

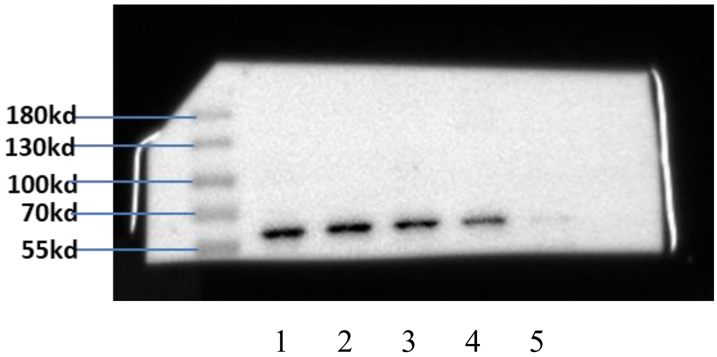

Figure S2 The WB raw photo of Vimentin in Figure 6. 1~5: NC, PTX, PTX+siAXL, PTX-NP, PTX-siAXL-NP

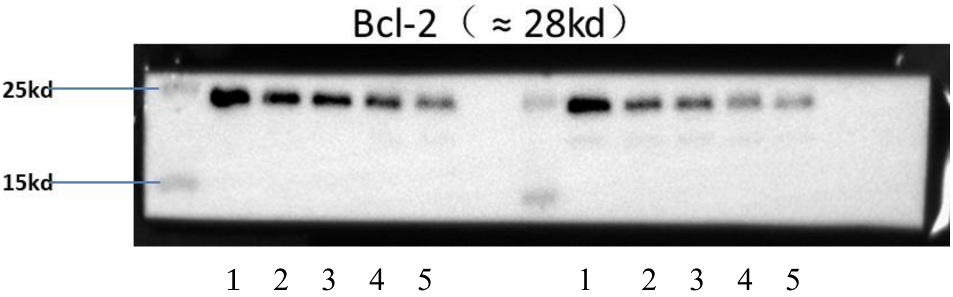

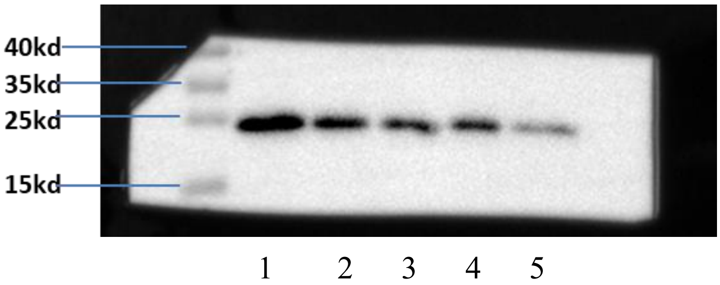

Figure S3 The WB raw photo of Bcl-2 in Figure 6. 1~5: NC, PTX, PTX+siAXL, PTX-NP, PTX-siAXL-NP

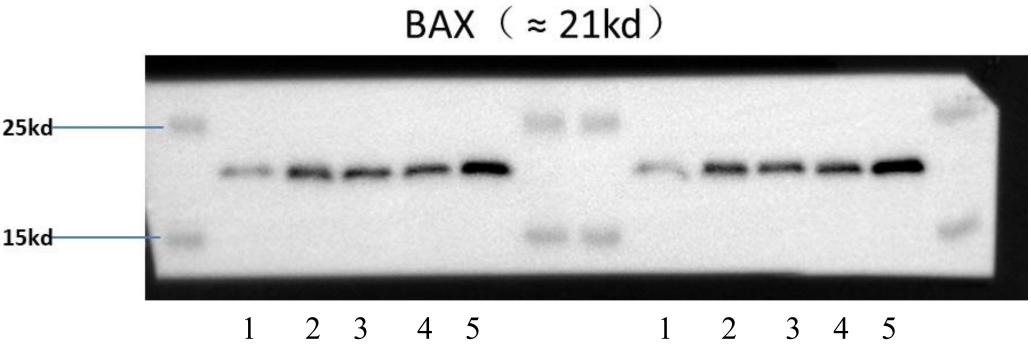

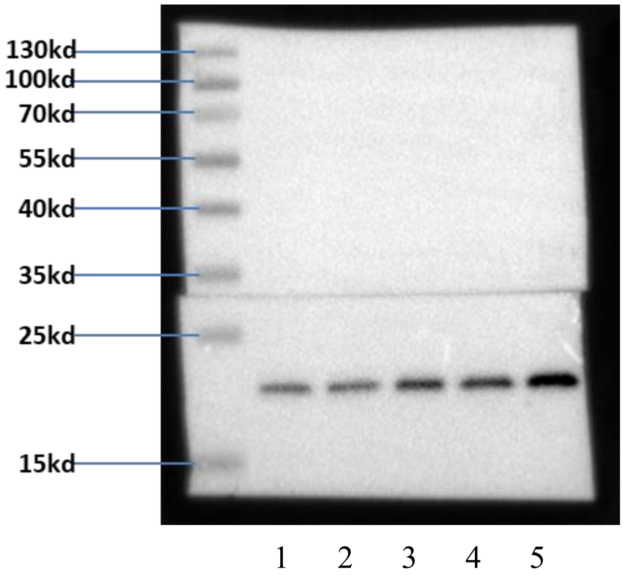

Figure S4 The WB raw photo of BAX in Figure 6. 1~5: NC, PTX, PTX+siAXL, PTX-NP, PTX-siAXL-NP

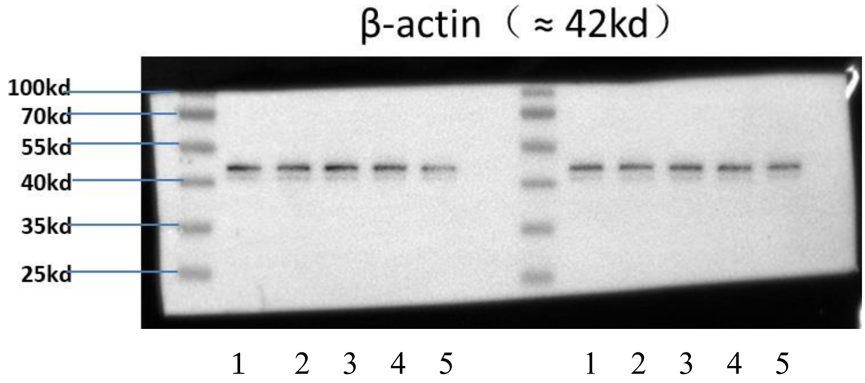

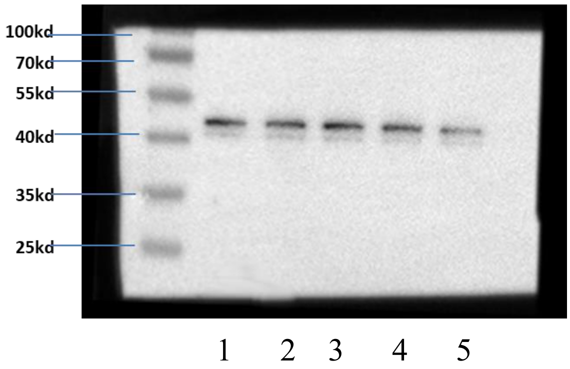

Figure S5 The WB raw photo of β-actin in Figure 6. 1~5: NC, PTX, PTX+siAXL, PTX-NP, PTX-siAXL-NP
